# Hyperpolarization-Activated Current Induces Period-Doubling Cascades and Chaos in a Cold Thermoreceptor Model

**DOI:** 10.3389/fncom.2017.00012

**Published:** 2017-03-10

**Authors:** Kesheng Xu, Jean P. Maidana, Mauricio Caviedes, Daniel Quero, Pablo Aguirre, Patricio Orio

**Affiliations:** ^1^Centro Interdisciplinario de Neurociencia de Valparaíso, Universidad de ValparaísoValparaíso, Chile; ^2^Departamento de Matemática, Universidad Técnica Federico Santa MaríaValparaíso, Chile; ^3^Facultad de Ciencias, Instituto de Neurociencia, Universidad de ValparaísoValparaíso, Chile

**Keywords:** chaos, hyperpolarization-activated current, conductance-based model, bursting, period doubling

## Abstract

In this article, we describe and analyze the chaotic behavior of a conductance-based neuronal bursting model. This is a model with a reduced number of variables, yet it retains biophysical plausibility. Inspired by the activity of cold thermoreceptors, the model contains a persistent Sodium current, a Calcium-activated Potassium current and a hyperpolarization-activated current (I_h_) that drive a slow subthreshold oscillation. Driven by this oscillation, a fast subsystem (fast Sodium and Potassium currents) fires action potentials in a periodic fashion. Depending on the parameters, this model can generate a variety of firing patterns that includes bursting, regular tonic and polymodal firing. Here we show that the transitions between different firing patterns are often accompanied by a range of chaotic firing, as suggested by an irregular, non-periodic firing pattern. To confirm this, we measure the maximum Lyapunov exponent of the voltage trajectories, and the Lyapunov exponent and Lempel-Ziv's complexity of the ISI time series. The four-variable slow system (without spiking) also generates chaotic behavior, and bifurcation analysis shows that this is often originated by period doubling cascades. Either with or without spikes, chaos is no longer generated when the I_h_ is removed from the system. As the model is biologically plausible with biophysically meaningful parameters, we propose it as a useful tool to understand chaotic dynamics in neurons.

## 1. Introduction

Chaotic behavior in neural systems has been observed for many years. Experimental observations of non-periodic responses range from molluscan neurons (Aihara et al., 1984) to rat sciatic nerves (Gu, 2013), including lobster CPGs (Abarbanel et al., 1996) and fish's Mauthner cells (Faure et al., 2000) (for a review, see Korn and Faure, 2003). In addition, chaotic behavior has been analyzed in detail in several models of neural excitability. Notable examples include the Plant model for the R15 bursting cell in Aplysia (Plant and Kim, 1976) that shows a chaotic regime between bursting and beating firing modes (Canavier et al., 1990); the Chay model of pancreatic β cells (Chay and Rinzel, 1985); and the Huber & Braun (H&B) model of cold thermoreceptors (Braun et al., 1998; Feudel et al., 2000).

Most of the models reported to show chaotic activity present different types of bursting oscillations, that arise from the interaction between fast membrane voltage dynamics and a slower current or intracellular mechanism, such as Calcium dynamics (Chay and Rinzel, 1985; Canavier et al., 1990; Falcke et al., 2000). Because of this evidence, the interaction of different time scales has been proposed as the origin of the chaotic behavior. A simpler model, of only 3 variables, that produces burst firing and is known as the Hindmarsh-Rose model (Hindmarsh and Rose, 1984), presents chaotic dynamics for some parameter combinations. Despite its simplicity, it shows a variety of aperiodic behaviors that are being actively studied by several groups (Holden and Fan, 1992; Abarbanel et al., 1996; Barrio and Shilnikov, 2011; Barrio et al., 2014), giving useful insight into the intrinsic mathematical mechanisms that drive bursting dynamics. However, a drawback of the Hindmarsh-Rose model is that some of its equations and parameters lack actual biophysical meaning, and thus its usefulness to interpret biological data is somewhat limited.

Instead, here we report and analyze the chaotic behavior of a bursting model inspired on the temperature-dependent firing patterns observed in cold thermoreceptors (Braun et al., 1980). Our model is derived from the H&B model (Braun et al., 1998), in which a slow membrane oscillation is driven by a mixed Sodium/Calcium current and a Calcium-activated Potassium current. Fast Sodium and Potassium currents produce action potentials, and the usual effect of temperature on channel kinetics makes the model to display different spiking patterns such as bursting, tonic, skipping and chaotic. Recently, a hyperpolarization-activated current (I_h_) was added to the model in order to reproduce and explain experimental results with genetic and pharmacological suppression of I_h_ (Orio et al., 2012). This extended model will be referred here as HB+Ih and it is the main object of study in this paper. We present here a parameter sweeping approach (Barrio and Shilnikov, 2011; Barrio et al., 2014) to explore the regions of chaotic behavior and its dependence on certain model parameters.

The original H&B model shows chaotic behavior at very low temperatures (<10° C), thus limiting the possible biological interpretations of this behavior. In contrast, we found that the new HB+Ih model displays chaotic behavior at physiological temperatures, namely in the 32–38°C range. Moreover, the chaotic behavior is highly dependent on the presence of I_h_ and its associated activation parameters; this is one of the most relevant features of the extended model. Importantly, we also show that chaos relies only in the slow oscillation subsystem, as chaos persists in the absence of the fast conductances that cause spiking. In addition, a bifurcation analysis shows that the transition from periodic to aperiodic behaviour—that is, from simple to chaotic dynamics—is organized by period doubling cascades (Guckenheimer and Holmes, 1983).

The manuscript is organized as follows: in Section 2 we describe the HB+Ih model and the numerical methods employed to analyze chaotic behavior. In Section 3, we present the results that include numerical simulations of the full system where we calculate different measures of chaos as we vary the system's parameters. Then we switch to the slow subsystem, doing systematic parameter explorations and bifurcation analysis. In Section 4 we summarize and discuss our findings.

## 2. Methods

### 2.1. Mathematical model

The basis of our model is the Orio et al. (2012) model that reproduces the static firing patterns of cold thermoreceptors. The equation for the membrane voltage *V* is:

(1) $$\begin{array}{l} C_{m}\frac{dV}{dt}=-I_{sd}-I_{sr}-I_{h}-I_{d}-I_{r}-I_{l}, \end{array}$$

where *C*_*m*_ is the membrane capacitance; *I*_*d*_, *I*_*r*_, *I*_*sd*_, *I*_*sr*_ are depolarizing (Na_V_), repolarizing (K_dr_), slow depolarizing (Na_P_ / Ca_T_) and slow repolarizing (K_Ca_) currents, respectively. *I*_*h*_ stands for hyperpolarization-activated current, and lastly *I*_*l*_ represents the leak current. Currents are defined as:

(2) $$\begin{array}{r} I_{i}=\rho \left(T\right)g_{i}a_{i}\left(V-E_{i}\right)\;i=d,r,sd,h,l; \end{array}$$

(3) $$\begin{array}{r} I_{sr}=\rho \left(T\right)g_{sr}\frac{a_{sr}^{2}}{a_{sr}^{2}+0.4^{2}}\left(V-E_{sr}\right), \end{array}$$

where *a*_*i*_ is an activation term that represents the open probability of the channels (*a*_*l*_ ≡ 1), with the exception of *a*_*sr*_ that represents intracellular Calcium concentration. Parameter *g*_*i*_ is the maximal conductance density, *E*_*i*_ is the reversal potential and the function ρ(*T*) is a temperature-dependent scale factor for the current. The activation terms *a*_*r*_, *a*_*sd*_, and *a*_*h*_ follow the differential equations:

(4) $$\begin{array}{l} \frac{da_{i}}{dt}=\phi \left(T\right)\frac{a_{i}^{\infty }\left(V\right)-a_{i}}{\tau_{i}}\;i=r,sd,h, \end{array}$$

where

(5) $$\begin{array}{l} a_{i}^{\infty }\left(V\right)=\frac{1}{1+\exp\left(-s_{i}\left(V-V_{i}^{0}\right)\right)}. \end{array}$$

On the other hand, *a*_*sr*_ follows

(6) $$\begin{array}{l} \frac{da_{sr}}{dt}=\phi \left(T\right)\frac{-\eta I_{sd}-\kappa a_{sr}}{\tau_{sr}}. \end{array}$$

Finally,

(7) $$\begin{array}{l} a_{d}=a_{d}^{\infty }=\frac{1}{1+\exp\left(-s_{d}\left(V-V_{d}^{0}\right)\right)}. \end{array}$$

The function ϕ(*T*) in Equations (4) and (6) is a temperature factor for channel kinetics. The temperature-dependent functions for conductance ρ(*T*) in Equations (2–3), and for kinetics ϕ(*T*) in Equations (4) and (6) are given, respectively, by:

(8) $$\begin{array}{l} \rho \left(T\right)=1.3^{\frac{T-25}{10}}\;\phi \left(T\right)=3^{\frac{T-25}{10}}. \end{array}$$

Unless stated otherwise, the parameters used are given in Table 1. Note that our set of equations presents some modifications from the original model in Orio et al. (2012). The first difference is that we do not consider the cold-inhibited trek current. This potassium current also contributes to the cold response as its inhibition produces depolarization of the cell (Viana et al., 2002; Noël et al., 2009). As the temperature dependence of the model is secondary to our objective, for simplicity reasons we omitted it. Secondly, and partially compensating the absence of the trek current, the leak current *I*_*l*_ is now temperature-dependent (as the rest of ionic currents) due to the ρ(*T*) temperature factor. We also want to stress a departure from the original H&B model, namely the introduction of a saturable *a*_*sr*_-dependent expression in equation 3. This modification, already introduced by Orio et al. (2012), is necessary in order to perform a meaningful parameter exploration. In the original H&B formulation, *a*_*sr*_ can grow far above 1 when *I*_*sd*_ is high, making the *g*_*sr*_ parameter no longer to be the *maximal sr* conductance.

**Table 1:** **Parameters of the HB+Ih model**.

**Parameter**	**Value**	**Units**
*C*_*m*_	1.0	μ*F*/*cm*^2^
*g*_*d*_	2.5	*mS*/*cm*^2^
*g*_*r*_	2.8	
*g*_*sd*_	0.21	
*g*_*sr*_	0.28	
*g*_*l*_	0.06	
*g*_*h*_	0.4	
$V_{d}^{0}$	−25	*mV*
$V_{r}^{0}$	−25	
$V_{sd}^{0}$	−40	
$V_{h}^{0}$	−85	
κ	0.18	–
η	0.014	*cm*^2^/μ*A*
τ_*r*_	2	*ms*
τ_*sd*_	10	
τ_*sr*_	35	
τ_*h*_	125	
*s*_*d*_	0.25	*mV*^−1^
*s*_*r*_	0.25	
*s*_*sd*_	0.11	
*s*_*h*_	−0.14	
*E*_*d*_,*E*_*sd*_	50	*mV*
*E*_*r*_,*E*_*sr*_	−90	
*E*_*l*_	−80	
*E*_*h*_	−30	

### 2.2. Numerical estimation of chaotic behavior

#### 2.2.1. Numerical calculation of maximal Lyapunov exponent for ordinary differential equations

The Lyapunov exponents give a measure of the exponential separation of nearby trajectories in a given direction (Guckenheimer and Holmes, 1983; Liu, 2010; Strogatz, 2014). In particular, a maximal Lyapunov exponent (MLE) greater than zero indicates sensitive dependence to initial conditions and, hence, is widely used as an indicator of chaos.

We calculated MLEs from trajectories in the full variable space, following a standard numerical method based on that of Sprott (2003) (see also Jones et al., 2009).

#### 2.2.2. Calculation of Lyapunov exponent from interval time series

In order to determine the Lyapunov exponent (LE) of the inter-spike interval (ISI) series, we proceeded as described in Kantz and Schreiber (2004). The method is based on Takens reconstruction theorem (Broer and Takens, 2011). Briefly, an ISI time series of length *n* is transformed into an *m*-dimensional reconstructed *R*_*m*_ phase space, in which every *k*-th state point is specified by a vector with *m* elements, each one of them taken from the original ISI time series:

$$\begin{array}{l} P_{k}=\left[ISI_{k},ISI_{k+1},ISI_{k+2},\ldots ,ISI_{k+m-1}\right],k=1,\ldots ,n-m+1. \end{array}$$

For a given state point *P*_*i*_ ∈ *R*_*m*_, we select all its neighbors $\left\{P_{i}^{*}\right\}$ within a certain vicinity of radius ϵ and we measure the mean Euclidean distance *d*_0_ from *P*_*i*_ to the elements in $\left\{P_{i}^{*}\right\}$. The value of ϵ is chosen and constantly adjusted so that a maximum of 0.05% of points in *R*_*m*_ fall within the vicinity. Next, the distances *d*_1_, *d*_2_, …*d*_*r*_ are calculated from the following points in the series *P*_*i*+1_, *P*_*i*+2_, …, *P*_*i*+*r*_ to the corresponding points that follow the elements in $\left\{P_{i}^{*}\right\}$, namely the sets $\left\{P_{i+1}^{*}\right\},\left\{P_{i+2}^{*}\right\},\ldots ,\left\{P_{i+r}^{*}\right\}$. The procedure is repeated for every single point in the series, thus obtaining a large number of distances *d*_0_, *d*_1_, …, *d*_*r*_.

Finally, we take the averages of the distances over all points 〈*d*_0_〉, 〈*d*_1_〉, …, 〈*d*_*r*_〉 and the LE is taken to be the slope of the plot log(〈*d*_*i*_〉) vs. *i*. We employed *r* = 6 and calculated LE for *m* (reconstructed dimension) = 7, 9 and 11. If for any value of *m* the regression yielded a *p*-value lower than 0.05 for the slope being different to 0, then we averaged the corresponding LE values. The number *r*, steps taken into account to advance the ISI series, and *m*, the embedding dimension, were empirically chosen by looking at the consistency of the results.

#### 2.2.3. Lempel-Ziv complexity estimation

Lempel-Ziv complexity estimation method is an approximation to the Kolmogorov and Martin-Löf definition (Lempel and Ziv, 1976). This uses the idea that a computer program—as it scans an *n*-word string *S* = *s*_1_*s*_2_ ⋯ *s*_*n*_ from left to right—adds a new word to its memory (or “vocabulary”) every time it discovers a sub-string of consecutive digits not previously encountered. The size of the vocabulary encountered and the rate at which new words are found along *S* are used in the Lempel-Ziv complexity measure. In this paper we are interested in the analysis of spike trains, thus to generate a binary sequence for a given spike-train it is necessary to divide the complete interval of measurement analysis in small sub intervals of size less than the minimum ISI and put one if there is a spike in the interval and zero if not.

Roughly speaking, the calculation of complexity is given by *c*(*n*)/*b*(*n*) where *b*(*n*) = *n*/log_2_*n* and *c*(*n*) counts the number of steps necessary to reconstruct the sequence *s*_1_*s*_2_ ⋯ *s*_*n*_ of size *n*. The procedure to find *c*(*n*) can be explained with the diagram given in Kaspar and Schuster (1987) and summarized as follows: the first digit *s*_1_ is always inserted to the vocabulary. Then, let *s*_*r*_ be the last digit of the sequence *S* that has been reconstructed. We consider *Q* = *s*_*r*+1_ and ask if *Q* is contained in the vocabulary of *S*. If *s*_*r*+1_ can be obtained by repeating elements from the vocabulary, we define a new *Q* = *s*_*r*+1_*s*_*r*+2_ and ask if it is in the vocabulary of *S* and so on until *Q* becomes so large that it cannot be obtained by copying a word from the vocabulary of *SQπ* (the operator π discards the last string added to *SQ*). Then, a new word is inserted into the vocabulary. *c*(*n*) is the number *c* of production steps to create a string, being the steps the vocabulary elements plus any repetition operation.

### 2.3. Bifurcation analysis

Equilibrium states and periodic solutions of a dynamical system may undergo critical transitions under parameter variation. These re-arrangements may result in drastic changes —known as bifurcations— of the global dynamics including the onset of chaos (Guckenheimer and Holmes, 1983; Broer and Takens, 2011; Strogatz, 2014). Among the most simple bifurcation phenomena that one can find are saddle-node or limit point (LP) bifurcations —characterized by the sudden birth or disappearance of two equilibrium points—; and a Hopf bifurcation (HB) —where a periodic orbit is born from an equilibrium.

For the purposes of this work, the so-called period doubling or flip bifurcation plays a crucial role to understand the transition to chaos. This bifurcation is characterized by the loss of stability of a periodic orbit of period, say *T*, and the simultaneous birth of a secondary periodic orbit with period ≈ 2*T*. This process may repeat itself many times under parameter variation —within a relatively small range of parameter values— in a phenomenon knows as a period doubling cascade. At each occurrence of a period doubling bifurcation within the cascade a new orbit emerges with approximately twice the period of the one that had been born at the previous bifurcation. The consequence of this mechanism is the existence of aperiodic (chaotic) dynamics for a range of parameter values at one end of the cascade bifurcation values; see (Guckenheimer and Holmes, 1983; Broer and Takens, 2011) and the references therein for more details. Today, different software packages allow one to detect and continue a given bifurcation in one or two control parameters. In this paper, we make use of XPPAUT (and the numerical routines within it) to carry on a careful, detailed computational bifurcation analysis of equilibria and periodic orbits.

### 2.4. Numerical simulation and analysis

For long simulations, to obtain large ISI sequences, the model was implemented and run in the Neuron simulation environment (Hines and Carnevale, 2001) and run from Python scripts (Hines et al., 2009). Typically, ISI sequences were obtained from 1,000 s of simulation after 30 s of equilibration that were discarded to remove transient behaviors. For MLE calculation, simulations were solved with a fourth-order Runge-Kutta scheme written in Python. No detectable differences were found between Python and Neuron simulations. Data analysis and plotting was performed with Python and the libraries Numpy, Scipy, and Matplotlib.

## 3. Results

### 3.1. Chaotic spiking in the HB+Ih model

**Figure 1:**
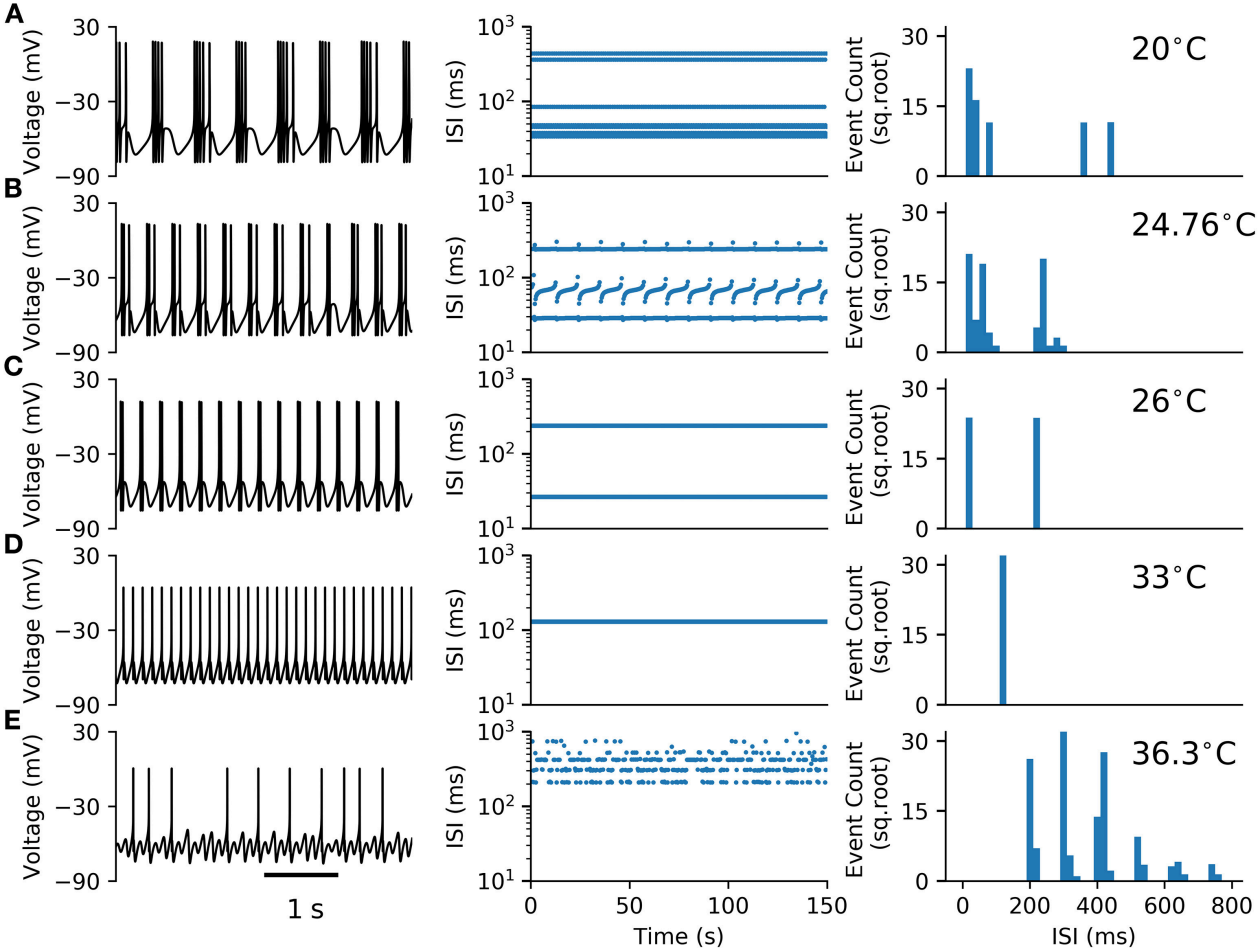
**Firing patterns observed in the HB+Ih model**. **(A–E)** Typical voltage time series (left), Inter-spike intervals (ISIs, middle) and ISI histograms (right) for the model at the shown temperatures. In **(A–D)** the histograms consider around 1100 spikes from 150 s simulations. In **(E)**, the histogram was built from 2977 spikes obtained in a 1000 s simulation.

The HB+Ih model (Equations 1–8) studied here is an extension of the Huber & Braun (H&B) model of cold thermoreceptor (Braun et al., 1998). To this model, a hyperpolarization-activated current (I_h_) was added in order to agree with experimental data obtained with I_h_ blockers and HCN1 knock-out mice (Orio et al., 2012). Like the original model, and reproducing the behavior of cold thermoreceptors under static temperature conditions, this model shows a variety of firing patterns as the temperature is changed. Figure 1 shows typical time series (voltage trace) of deterministic simulations of the model at five different temperatures. At 20, 24.76, and 26 °C the model displays a periodic bursting pattern, decreasing the number of spikes per burst as temperature increases. At 33 °C, periodic tonic firing is observed, and at 36.3 °C, the pattern becomes irregular with “skipping,” i.e., some oscillations do not generate a spike and thus the intervals are distributed in a polymodal fashion. The irregular firing, evidenced in the ISI plot and the ISI histogram at 36.3 °C, suggest a typical chaotic dynamic.

**Figure 2:**
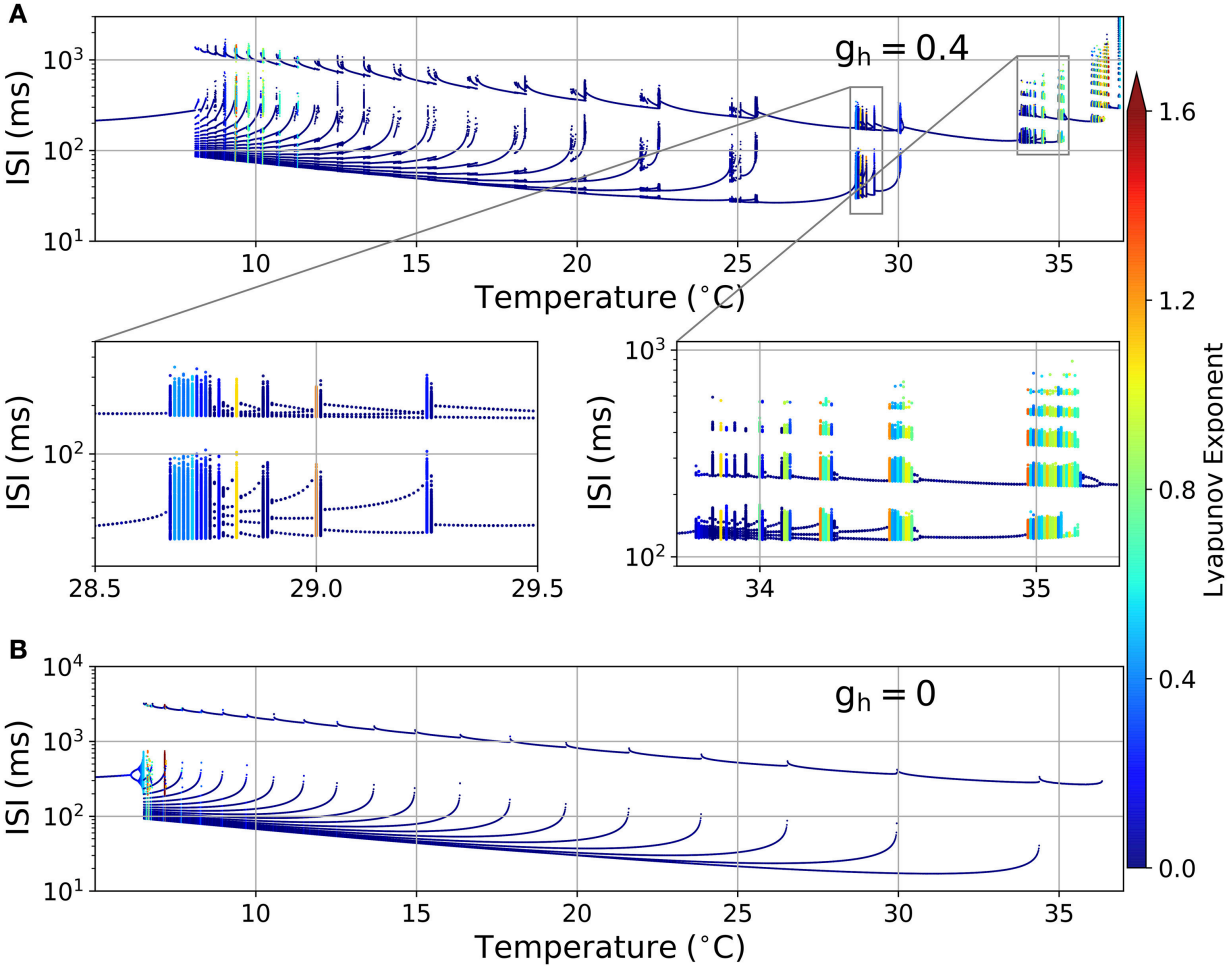
**(A)** ISI bifurcation plot of the HB+Ih model as temperature is changed. Each temperature was independently simulated (from 0 to 40 °C in 0.01 °C intervals), and the spikes were considered to occur each time the voltage crossed a −15 mV threshold. The color of the dots represents the Lyapunov exponent calculated from the ISI series. Two sections are shown below in expanded horizontal scale. **(B)** ISI bifurcation plot of the model in the absence of *I*_*h*_ (*g*_*h*_ = 0).

Figure 2A shows an ISI bifurcation plot against temperature. Visual inspection shows a high multiplicity of ISI values at almost every transition between firing modes: around 35 °C when skipping patterns appear (right zoom), around 29 °C when bursting occurs (left zoom) and then each time a new spike is added to the bursting pattern. This multiplicity of intervals suggests an irregular firing which is characteristic of chaotic behavior. However, calculation of the Lyapunov Exponent (LE) from the ISI time series (color code in Figure 2) reveals that not all the spike patterns that have a large number of ISI values are chaotic. Some of them, like the pattern at 24.76 °C in Figure 1B, have a large number of ISI values but still are highly repetitive and thus display a LE value near 0. We designate these firing patterns as “complex” but not chaotic. There are also some chaotic firing patterns around 10 °C, near the transition to the tonic firing behavior, like the original H&B model, which display chaos only between 7 and 12 °C (Feudel et al., 2000). However, the H&B model does not display chaotic dynamics at higher temperatures, where the model becomes physiologically more relevant. We suspect that chaos at high temperatures is introduced by the presence of the I_h_, and this is confirmed in Figure 2B where our model is simulated in the absence of I_h_ (*g*_*h*_ = 0). This diagram looks different to what has been described for the original H&B model (Feudel et al., 2000), because of some differences in parameters and the use of a saturable function of *a*_*sr*_ in the *I*_*sr*_ expression (equation 3). However, important qualitative features are conserved: no chaos (nor complex firing patterns) is observed above 10 °C, and chaotic firing patterns are only seen near the transition to tonic firing at low temperatures. This means that chaos at high temperatures is mainly introduced by I_h_ and not by the other minor modifications that were made to the model (see Section 2.1).

Although the HB+Ih model was inspired in the firing patterns of cold thermoreceptors at different temperatures, the combination of ion currents in this model is not exclusive to sensory neurons. The dynamics of this model can resemble other neurons in the CNS, where temperature changes are of lesser importance. Thus, we decided to study how the chaotic dynamics of the model depends on other parameters and the rest of the work presented here was performed with the temperature fixed at 36 °C, very close to the physiological value.

### 3.2. Chaotic behavior in the full system and the role of slow conductances

**Figure 3:**
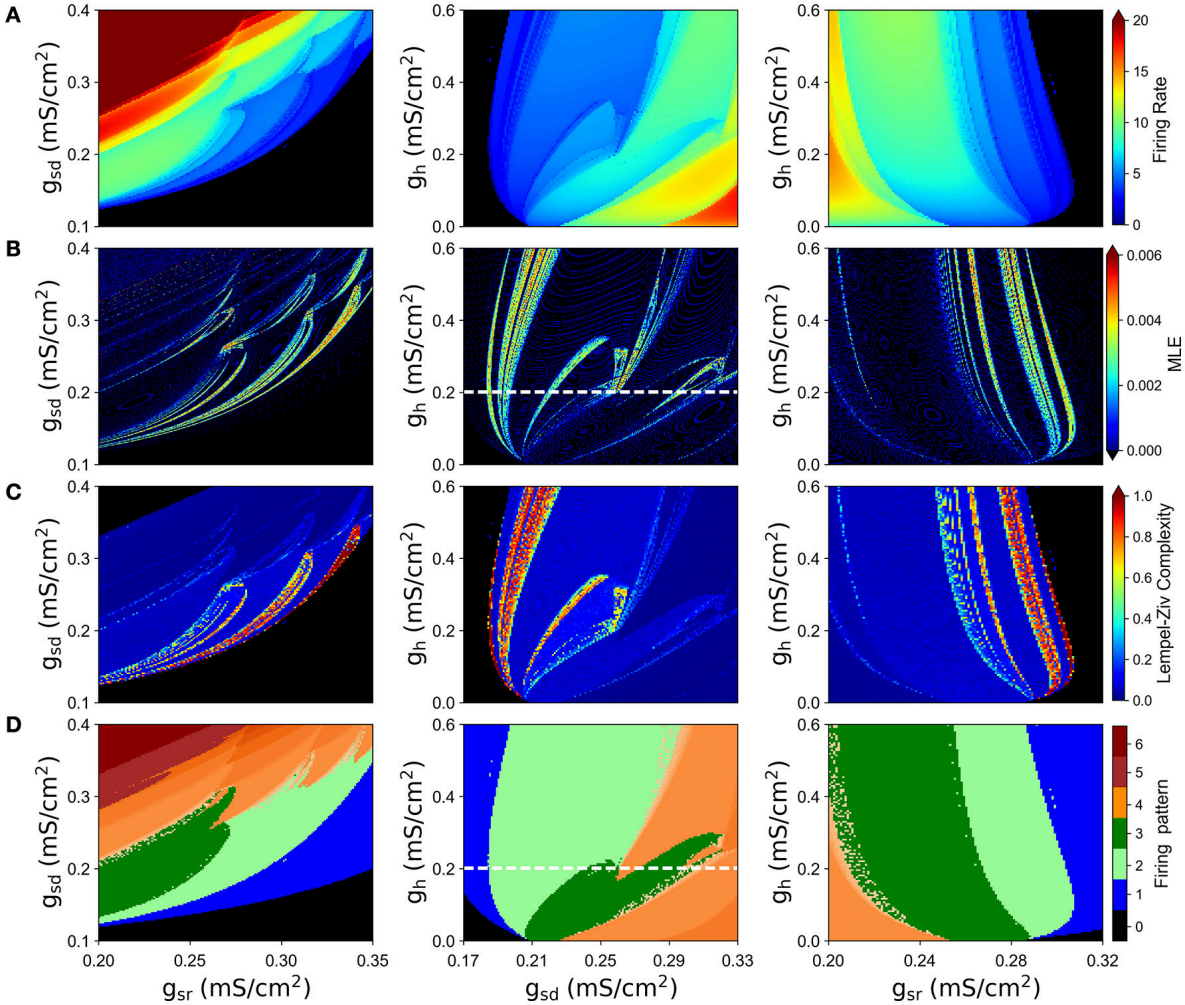
**Chaotic behavior of the model at different combination of the slow conductance densities ***g***_***sd***_, ***g***_***sr***_ and ***g***_***h***_**. **(A)** Firing rate in spikes/s. **(B)** MLE of the voltage trajectories. **(C)** Lempel-Ziv complexity. **(D)** Firing pattern. The color bar indicates: 0, no oscillations; 1, sub-threshold oscillations (no spikes); 2, oscillations and spikes with skipping; 3, regular tonic spiking; 4, burst firing (the shade represents the number of spikes per bursts); 5, tonic with firing rate between 20 and 50 spikes/s; 6, firing rate higher than 50 spikes/s.

We simulated the model with different combinations of the slow currents maximal densities *g*_*sd*_, *g*_*sr*_, and *g*_*h*_, and calculated the Lyapunov exponent of the ISI time series and the maximum Lyapunov exponent (MLE) from the voltage trajectories. As an alternative measure of chaotic behavior, we calculated the Lempel-Ziv complexity of the ISI data which has been used previously to prove the existence of chaos in neural models (Xu et al., 1997; Lu et al., 2008; Yang et al., 2009) and other disciplines (Frank and Stengos, 1988; Lu et al., 2008). MLE and complexity measures are shown in Figures 3B,C, respectively, together with the mean firing rate (average spikes per second) in Figure 3A and the firing pattern (bursting, tonic, skipping or no firing) in Figure 3D. In particular, Figure 3A shows that most of the explored region in parameter space corresponds to firing rates below 10 spikes/s. Though MLE (Figure 3B) and Complexity (Figure 3C) results are not completely overlapping, a high degree of correspondence can be seen on the results. Moreover, LE from ISIs mostly agrees with the results of MLE (not shown in Figure 3 but see for instance **Figure 6A**). Chaotic behavior is concentrated in regions with low (<5) firing rate mostly, where skipping or polymodal firing pattern occurs. There is also chaotic firing at higher firing rates, but in narrower regions of the parameter space.

**Figure 4:**
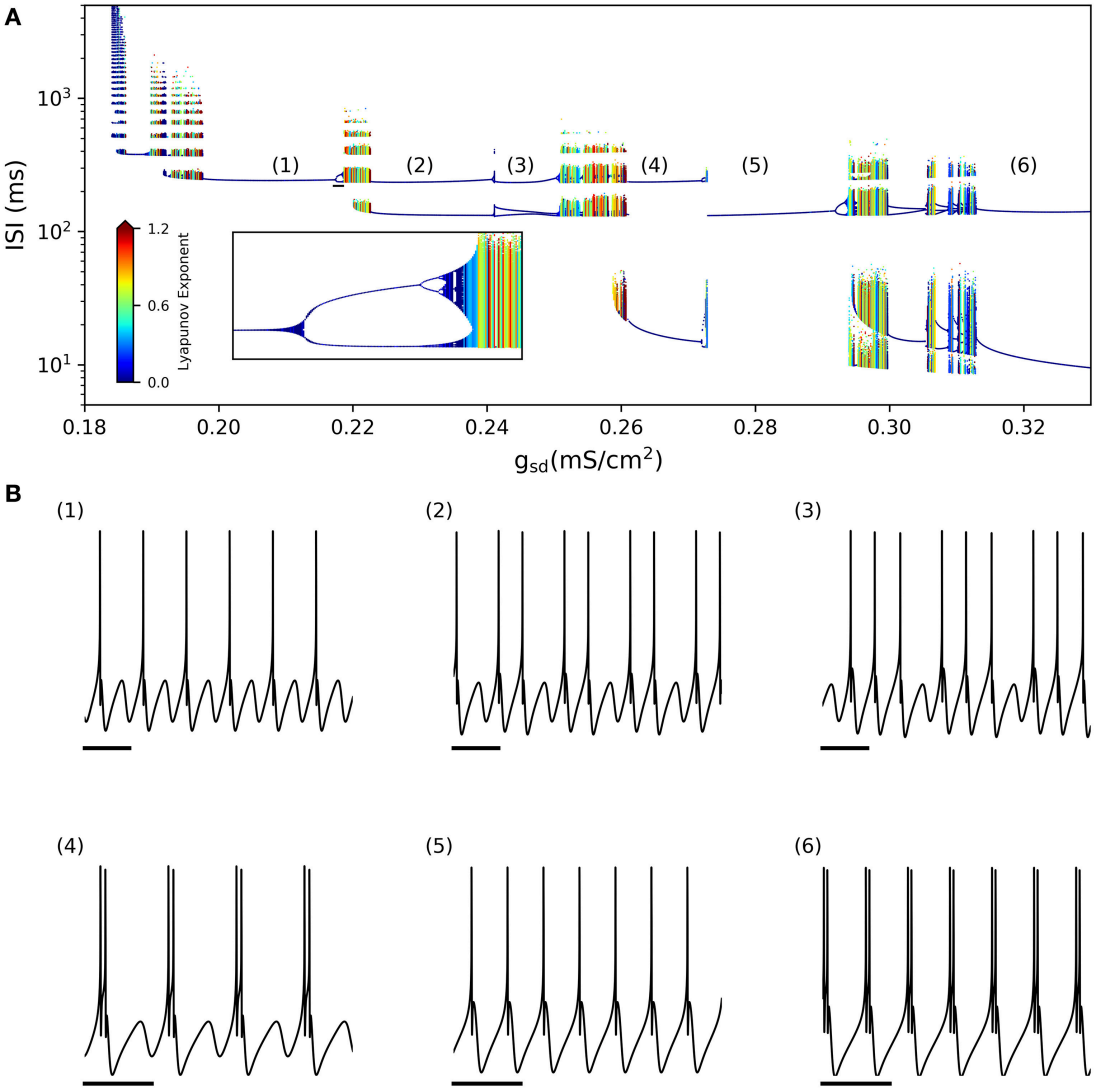
**(A)** ISI bifurcation of the model as the *g*_*sd*_ parameter is changed and *g*_*h*_ is fixed to 0.2 mS/cm^2^. Numbers denote different firing modes between which chaotic regions are found. Inset shows a detail of the ISI-doubling events to the right of region (1), from *g*_*sd*_ = 0.217–0.219. (small black bar in the large plot) **(B)** Sample voltage trajectories showing the firing modes. (1), (2), and (3) correspond to different skipping patterns; (4) is bursting with skipping; (5) is regular tonic; and (6) is regular bursting. In all traces the scale bar is 250 ms.

By looking at the center column of Figure 3 (*g*_*sd*_ vs. *g*_*h*_), we note that as *g*_*sd*_ increases the system alternates between several firing patterns and it exhibits chaotic firing in a vicinity of almost every transition. To illustrate this better, we selected *g*_*h*_ = 0.2 (white dashed line in B and D) and performed an ISI bifurcation diagram on the parameter *g*_*sd*_. Figure 4A shows that the model displays several firing modes and most (if not all) of the transitions between them imply a chaotic behavior. We find noteworthy the transitions between different skipping (polymodal) firing patterns —three of which are shown in Figure 4B—, and a transition between “skip-bursting“ and regular tonic modes (denoted as (4) and (5), respectively). Figure 4 also shows how these chaotic transitions that separate different firing modes are created. Take for instance firing mode (1) where the ISI value is kept almost constant for a relatively large range of *g*_*sd*_ values. As parameter *g*_*sd*_ becomes greater than a certain critical value (inset), there are two possible ISI values. This duplication of ISI values continues as *g*_*sd*_ is further increased, giving rise to what can be effectively understood as a cascade of “ISI doublings” —very much like in a period-doubling scenario. We confirmed this by repeating the plot at a much higher resolution (i.e. more values of *g*_*sd*_, separated by 10^−5^*mS*/*cm*^2^) (inset). It is important to recall that each value of *g*_*sd*_ was independently simulated, so there is no possible effect of the direction of parameter change; we also inspected the critical ISI time series to check that there were no transients involved in the results, thus ruling out an artifact due to initial conditions.

**Figure 5:**
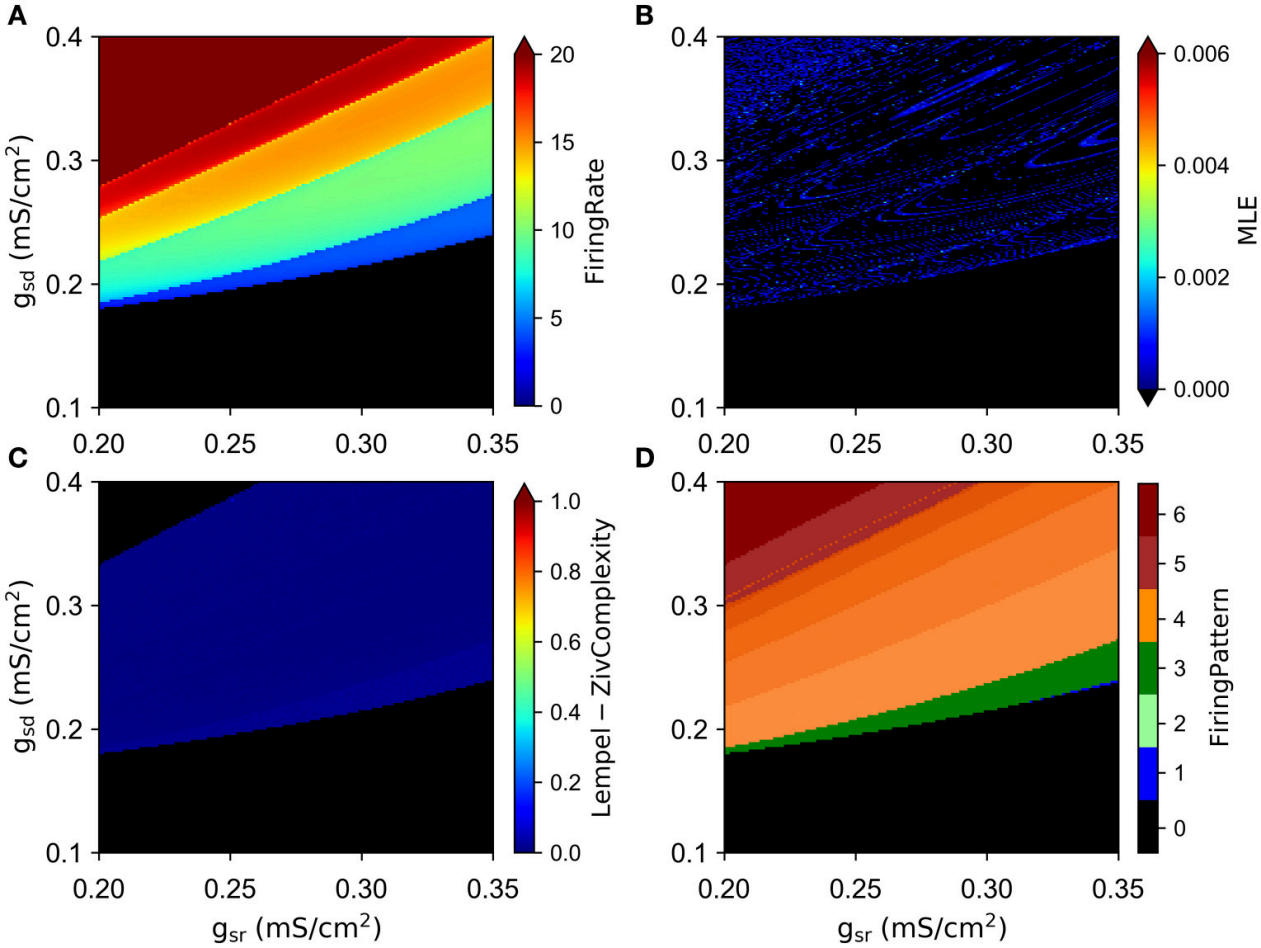
**Absence of chaos in the model when ***g***_***h***_ = 0**. **(A–D)** are as described in Figure 3.

The limit behaviour where *g*_*h*_ = 0 can readily be seen in the center and right columns of Figure 3. This visual inspection suggests that in the absence of *I*_*h*_ there is no chaotic behavior. To test this idea, we explored again the (*g*_*sd*_, *g*_*sr*_) parameter subspace but now considering *g*_*h*_ = 0 in Figure 5. The calculations clearly show that, in the absence of *I*_*h*_, no chaotic behavior is detected, even though similar firing rates and firing patterns are produced.

**Figure 6:**
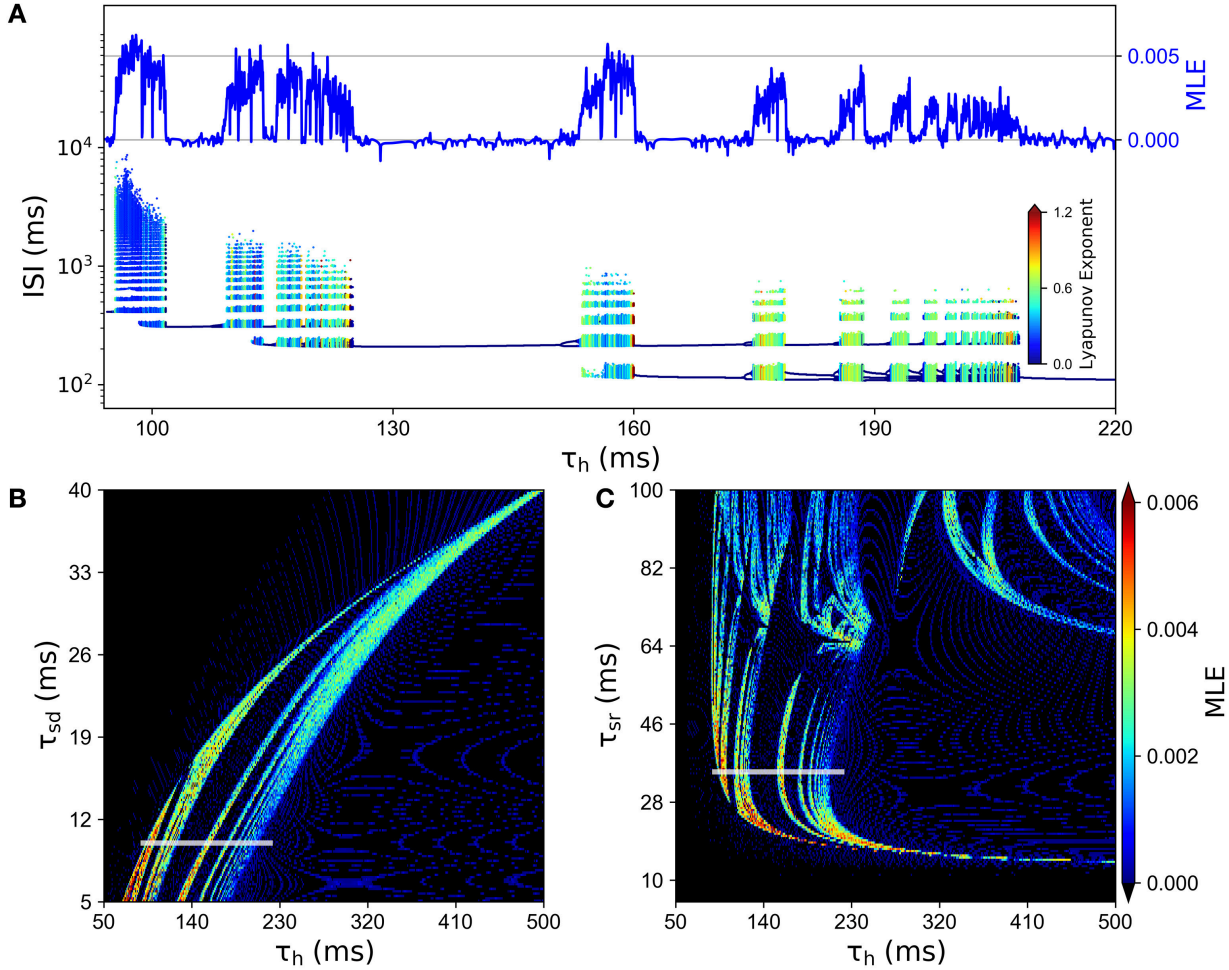
**Chaotic behavior dependency on the time constants of slow conductances τ_***sd***_, τ_***sr***_ and τ_***h***_**. **(A)** ISI bifurcation diagram as τ_*h*_ is changed. Color of ISIs represents the LE of the ISI sequence. At the top, the line depicts the MLE of the voltage trajectories for the same values of τ_*h*_. **(B)** MLE of voltage trajectories for different combinations of τ_*h*_ and τ_*sd*_. **(C)** MLE for different combinations of τ_*h*_ and τ_*sr*_. In **(B,C)**, the white line indicates the corresponding region of the parameter space that is explored in **(A)**.

In order to better characterize the involvement of *I*_*h*_ in the chaotic behavior of the model, we explored how the system depends on the time constant for this slow current, namely the parameter τ_*h*_. Figure 6A shows an ISI bifurcation diagram against τ_*h*_, showing that indeed the chaotic behavior depends on this parameter. In particular, the chaotic features disappear when the time constant is above 210 ms. In this Figure, we also show the good correspondence between the MLE calculated from voltage traces (top) and the LE calculated from the ISI sequences (see color code). Figures 6B,C show that the chaotic behavior depends on all the slow time constants, τ_*h*_, τ_*sr*_ and τ_*sd*_.

### 3.3. The chaotic behavior in the slow subsystem

**Figure 7:**
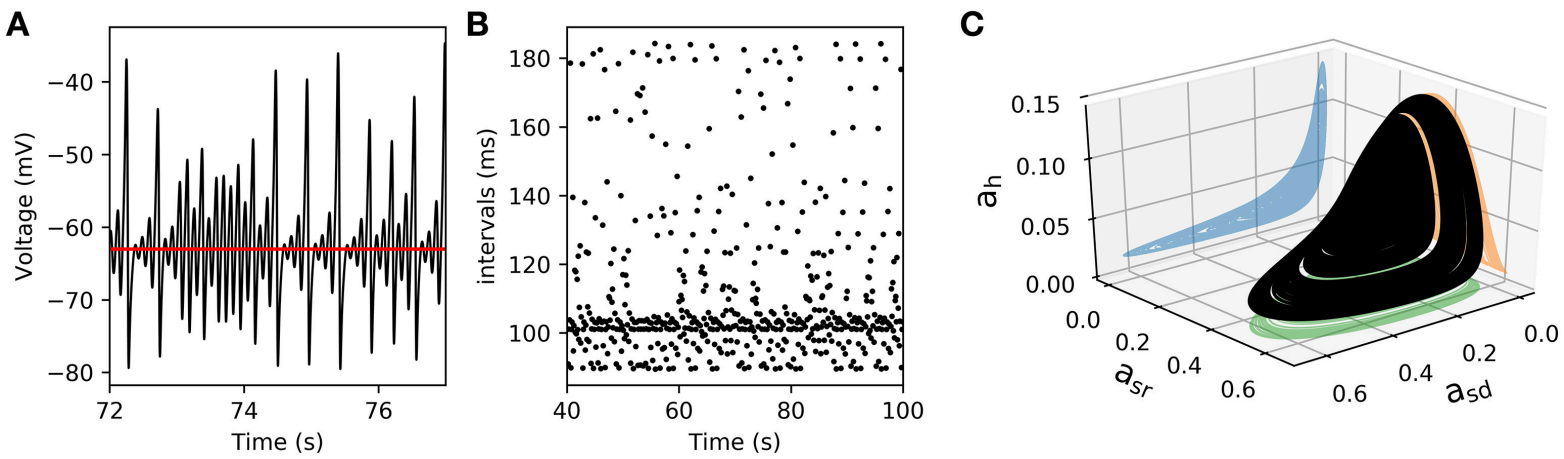
**Chaotic behavior in the slow subsystem of the model**. **(A)** Voltage trajectory of the model with *g*_*d*_ = *g*_*r*_ = 0, and $g_{sd}=0.222\left(mS/cm^{2}\right)$. The rest of parameters are as given in Table 1. The red line depicts *V*_*eq*_, the value of *V* at the unstable singular point associated to the attractor. **(B)**, Time intervals between successive crossings (with positive slope) of the *V*_*eq*_ value. **(C)**, 3D plot of a strange attractor in the *a*_*sd*_, *a*_*sr*_, *a*_*h*_ sub-space (black trace). The 2-D projections onto the corresponding planes are shown in color.

**Figure 8:**
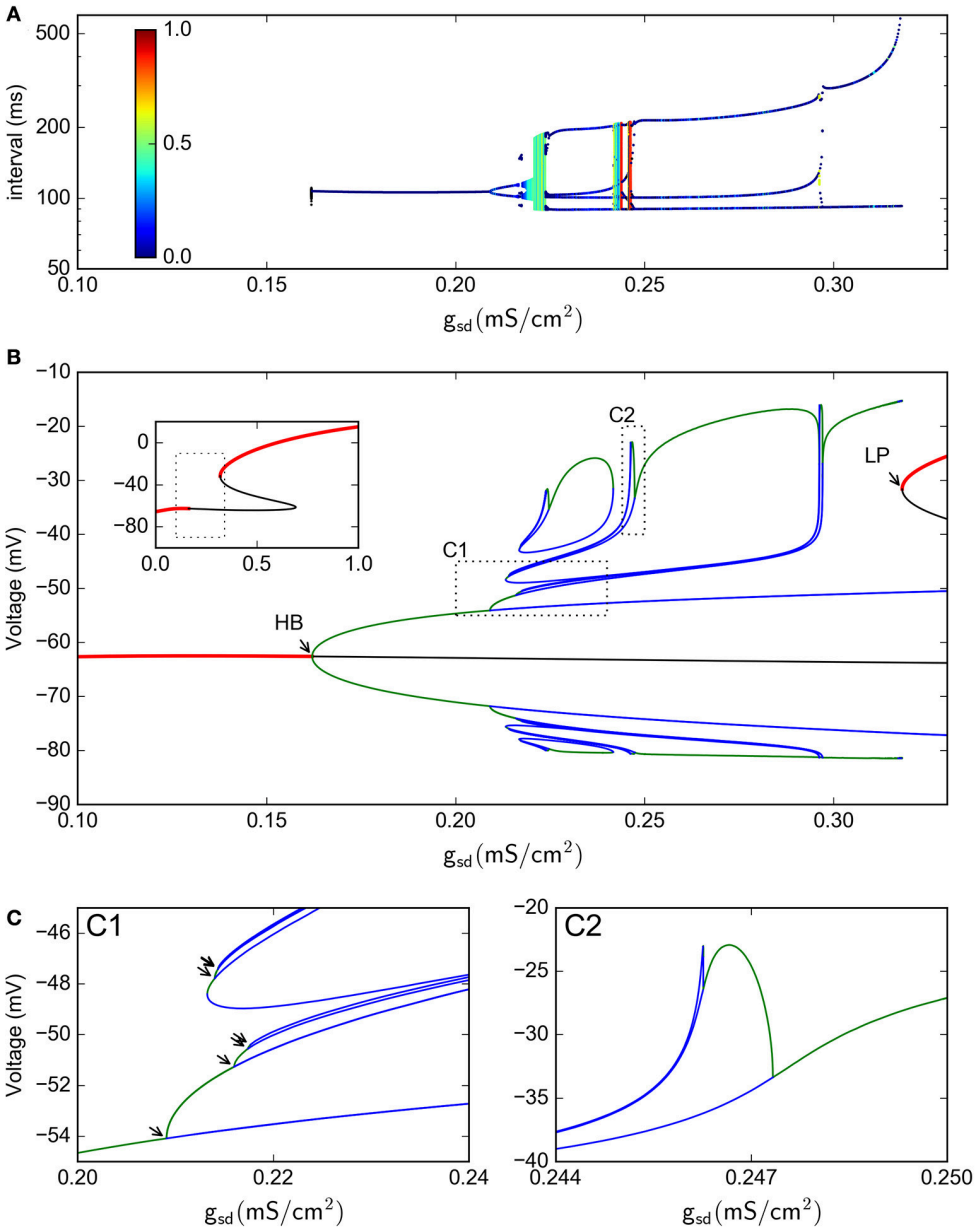
**Bifurcation analysis of the slow subsystem**. **(A)** Intervals between *V*_*eq*_ crossings at different *g*_*sd*_ values. The colors represent the LE of the interval sequence. **(B)** Bifurcation of the system, as calculated by continuation methods in XPPAUT. Red lines represent stable fixed points; black line is an unstable fixed point. Green lines are maxima and minima of stable periodic atractors, blue are maxima and minima of unstable periodic attractors. The inset shows only the stable/unstable fixed point in a wider *g*_*sd*_ range. HB = Hopf bifurcation, LP=Limit point. **(C)**, zoom of the C1 and C2 regions shown in **(B)**. In C1, the arrows show period doubling events. In this Figure, *g*_*h*_ = 0.4

We further reduced the model by eliminating the fast spike mechanism and leaving only the slow oscillation mechanism. In other words, we take the instantaneous variable *a*_*d*_(*t*) ≡ 0 and the fast recovery variable *a*_*r*_(*t*) ≡ 0, which is the same as setting the parameters *g*_*d*_ = *g*_*r*_ = 0. In this way, the system now reduces, effectively, from five to four dimensions. As shown in Figure 7, the model still retains a chaotic behavior, showing a complex oscillatory pattern. Computations also show that —for certain parameter values— the solutions of interest converge in the long term to a strange attractor which is shown in Figure 7C in a projection onto the subspace of the *a*_*h*_, *a*_*sr*_, and *a*_*sd*_ variables. To characterize this system, Figure 7B shows a return interval map (measured in *ms*) considering the voltage at the equilibrium point as a threshold (red line in Figure 7A). Figure 8A shows a bifurcation diagram that depicts the dependence of these return intervals on parameter *g*_*sd*_; in addition, the color scale shows the corresponding LE measures. Note that chaotic regions are preceded —as *g*_*sd*_ is increased— by doublings of return intervals very much like in Figure 4A.

With this 4-dimensional reduced system we were also able to perform a bifurcation study of equilibrium points and periodic orbits, shown in Figure 8B for the voltage vs. the *g*_*sd*_ parameter (the inset shows only the fixed points in a wider *g*_*sd*_ range). This bifurcation diagram shows the birth of a stable periodic orbit at a Hopf bifurcation (labelled as HB). As *g*_*sd*_ increases, this primary periodic orbit becomes the germ of a period doubling cascade (indicated by arrows in the enlargement in Figure 8C). This sequence of period doubling transitions coincides with the generation of chaotic regions identified in the return intervals plot. Note that only a limited number of period doubling branches were calculated and are shown here, because of space constraints. The period doubling events kept appearing as more branches were followed in the bifurcation. The chaotic regime is pulled back by a “reversed” period doubling mechanism as *g*_*sd*_ is further increased; two of these bifurcations are illustrated here in inlet C2. Finally, for *g*_*sd*_ > 0.3, a single stable periodic orbit exists. The oscillations eventually disappear at the *g*_*sd*_ value corresponding to label LP which coincides with a saddle-node or limit point bifurcation of equilibria; this phenomenon is known as an infinite-period bifurcation or saddle-node homoclinic point (Aguirre, 2015). This combination of homoclinic phenomena and period-doubling cascades has been also reported and described as one of the mechanisms that produce chaos in the Hindmarsh-Rose equations (Linaro et al., 2012; Barrio et al., 2014, 2017).

**Figure 9:**
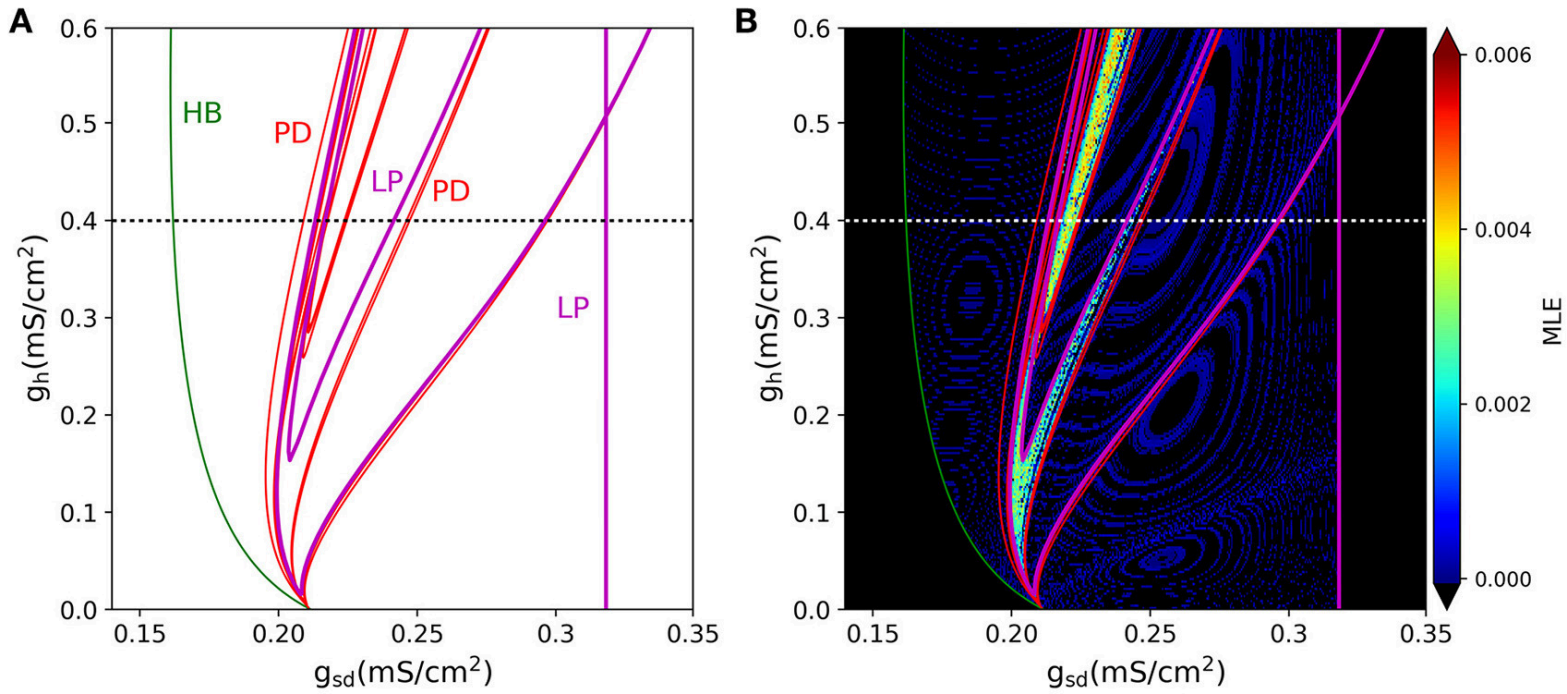
**(A)** Two-D parameter bifurcation of the slow subsystem. The HB, LP and PD (period doubling) points identified in Figure 8B are followed as *g*_*sd*_ and *g*_*h*_ change. Note that the PD curves do not touch the *g*_*h*_ = 0 axis. **(B)**, The bifurcation curves are superimposed to the MLE calculated from voltage trajectories of the same system, to show that PD cascades delimit the chaotic regions. Dotted lines in **(A,B)** (black and white, respectively) refer to the parameter region explored in Figure 8.

A 2-parameter bifurcation diagram for the slow system is shown in Figure 9A. In this plot, we see how the period-doubling points, the Hopf bifurcation, and the Limit-Point bifurcation that ends the oscillation extend as bifurcation curves as both parameters *g*_*sd*_ and *g*_*h*_ are allowed to vary. As the maximal conductance *g*_*h*_ is increased, more period doubling curves are added, enlarging the region of parameter values that allow chaos. Figure 9B shows the same bifurcation curves superimposed to the MLE values calculated from voltage trajectories. The resulting picture is revealing in that it shows how the regions of higher MLE fit perfectly into the predicted limits of chaotic dynamics generated by the period doubling phenomena. Hence, these findings emerge as another strong evidence to point out I_h_ as the main responsible for the chaotic behavior of the model.

**Figure 10:**
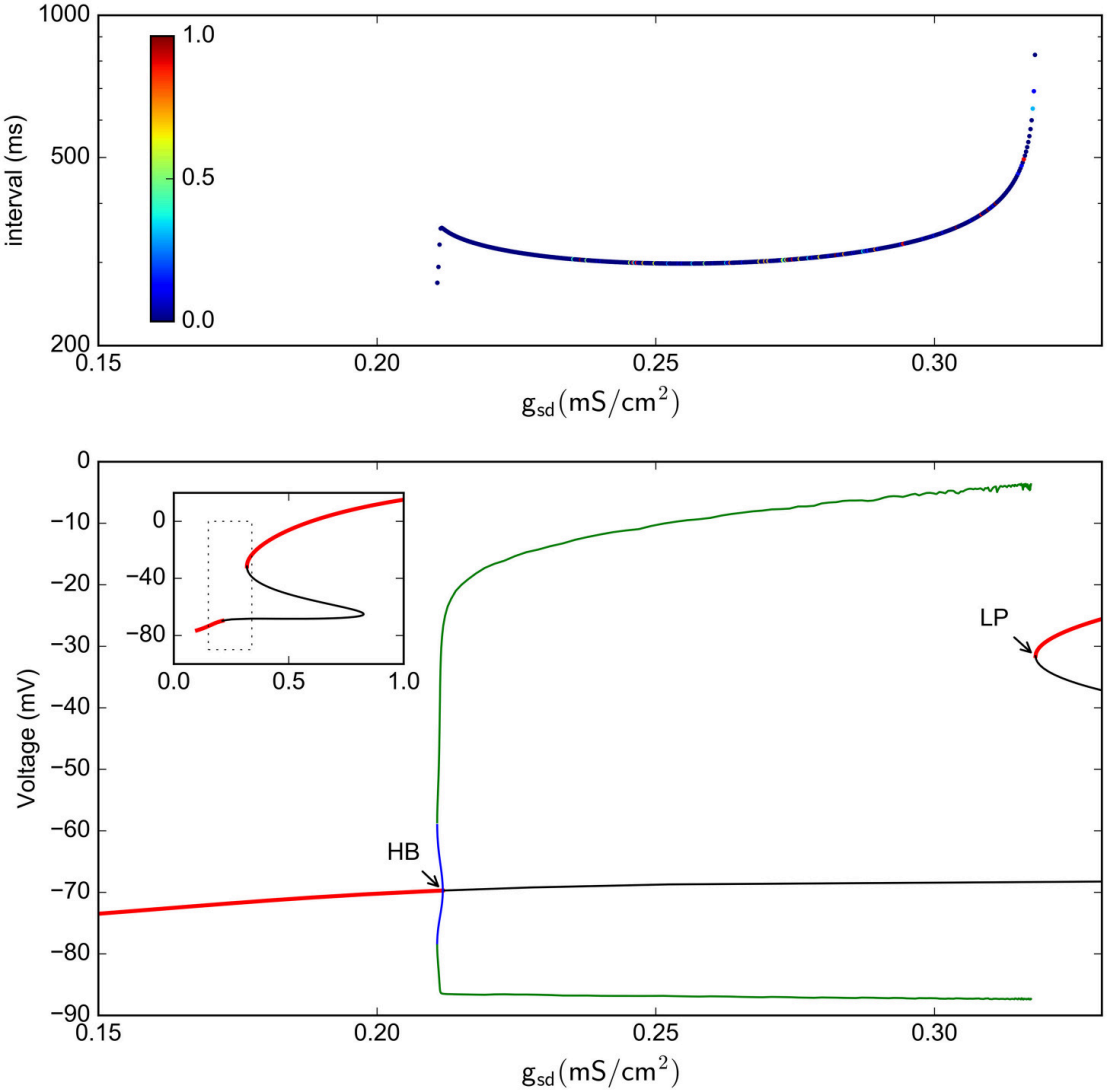
**Bifurcation analysis of the slow subsystem in the absence of I_**h**_ (***g***_***h***_ = 0)**. **(A)** Intervals between *V*_*eq*_ crossings at different *g*_*sd*_ values. The colors represent the LE of the interval sequence. **(B)** Bifurcation of the system, as calculated by continuation methods in XPPAUT. Red lines represent stable fixed points; black line is a unstable fixed point. Green is maxima and minima of stable periodic atractors. The inset shows only the stable/unstable fixed point in a wider *g*_*sd*_ scale. HB = Hopf bifurcation, LP = Limit point.

This result is further enforced by the realization that —as with the full (spiking) system— there is no chaos in the absence of I_h_. Indeed, the period doubling bifurcation curves actually do not touch the *g*_*h*_ = 0 axis. This fact becomes evident when the 1-parameter bifurcation is done with *g*_*h*_ = 0. Figure 10 shows that this system with *g*_*h*_ = 0 indeed has no period doubling events, retaining only the Hopf and Limit-Point bifurcations.

## 4. Discussion

In this article we have shown that a conductance-based model (denoted as HB+Ih model) displays chaotic behavior in many biologically plausible regions of the explored parameter space. These results are based on different numerical techniques to uncover and try to explain the regions of chaotic oscillations. To show the presence of chaos in a quantitative way, we have measured the maximal Lyapunov exponent of the system for different parameter scenarios, the Lyapunov exponent of the inter spike interval series and estimated the Lempel Ziv complexity. Bifurcation analysis on the reduced model without spikes allowed us to identify several period doubling cascades and propose them as the mathematical mechanism that originates chaos.

We discovered that the appearance of chaotic dynamics is related to the presence of a hyperpolarization-activated current (I_h_), commonly found in neurons throughout the Central Nervous System (Biel et al., 2009; He et al., 2014) and that plays important roles in rhythmic firing, also controlling the membrane potential and regulating synaptic plasticity. The other distinctive elements of the model resemble persistent Sodium currents (INa, P) and Calcium-activated Potassium channels (IK, Ca), also found in neurons that generate oscillatory rhythms (Llinás, 1988; Sanhueza and Bacigalupo, 2005). Thus, this model can serve as a general framework for studying how the interactions between different ion currents can generate chaotic oscillations.

The HB+Ih model offers the advantage of having a few number of variables while its equations and parameters are biophysically meaningful. In particular, chaos is observed in a 4-dimensional reduced model that considers the slow variables only. Unstable orbits and chaotic dynamics have been experimentally described in thermoreceptors (Braun et al., 1999a,b). The original H&B model, however, only exhibits such complex dynamics at around 8°C, far from the physiological range (Feudel et al., 2000). The HB+Ih model, on the other hand, readily displays irregular and chaotic dynamics at temperatures above 30° C, and thus it may be a more suitable system to explain the dynamics of cold thermoreceptors. Most of the parameter explorations presented here are based on the maximum conductance densities, that reflect the constantly changing levels of ion channel expression. Thus, the variety of firing patterns that we found here and the chaotic transitions between them are expected to be found under physiological conditions.

Some adjustments incorporated by Orio et al. (2012) (compared to the original H&B model Braun et al., 1998) made a further shift toward biological plausibility. These changes include a slightly higher voltage dependence of the INa, P – in accordance to what has been measured in somatosensory neurons (Herzog et al., 2001). Also, in the original H&B model the *I*_*sr*_ current depends linearly on *a*_*sr*_, a variable that is not naturally bound by the model. Therefore, in a parameter space exploration where *I*_*sd*_ can increase to high levels (because of a high *g*_*sd*_ value), *a*_*sr*_ would follow it to values much higher than 1, then losing its meaning as a channel open probability. In contrast, in the HB+Ih model, this term was replaced by a saturable binding term (see Equation 3) that allows the model to remain meaningful at high values of *g*_*sd*_.

Under the variation of parameters, even minimal 3-variable models of bursting neurons exhibit a rich variety of periodic and aperiodic dynamical patterns corresponding to different spiking and bursting regimes. The transitions between these patterns may contain complex dynamical structures such as period-doubling (PD) cascades and deterministic chaos. For instance, it has been reported that transition mechanism from tonic spiking to bursting in a class of bursting neurons (square-wave bursters) is based on periodic spiking with a series of period-doubling bifurcations followed by a homoclinic bifurcation of a saddle equilibrium (Terman, 1992; Wang, 1993; Feudel et al., 2000). Moreover, chaos has been proposed as a key signature for the transition between bursting and tonic firing (Chay, 1985; Rinzel and Ermentrout, 1989; Canavier et al., 1990; Terman, 1992; Feudel et al., 2000) (Terman (1992) gives a rigorous proof of the existence of Smale horseshoes). Then it has been shown that chaotic spiking can be generated close to the transition from spiking to bursting through period-doubling cascades (Medvedev, 2006). In our model, chaotic regimes have been detected in regions at the transitions between different types of oscillations. The first region of chaos appears through the transition from subthreshold oscillations (with no spikes) to irregular spiking (also called polymodal firing or skipping). This chaos appears through a cascade of Period Doublings, unrelated to the Hopf bifurcation that causes the appearance of subthreshold oscillations. In fact, when exploring different combination of *g*_*sd*_ and *g*_*h*_, the range of *g*_*sd*_ exhibiting subthreshold oscillation with no spikes become widened as *g*_*h*_ increases, thus separating the Hopf bifurcation from the chaotic region. Even though at smaller *g*_*h*_ the onset of chaos gets closer to the bifurcation, they seem to be unrelated. A second region of chaos emerges via periodic spikes to aperiodic spiking transition. These two kinds of transitions from periodic to aperiodic oscillations have been described as a mechanism that triggers chaotic spiking action potentials via period-doubling bifurcations. In the higher firing rate regimes, the HB+Ih model generates the third type of chaos at the transition from skipping to burst firing. In this case, the HB+Ih model exhibits chaotic bursting.

The period-doubling scenario was generally detected at one boundary of the chaotic regions (for instance, increasing *g*_*sd*_ but not decreasing), being the other boundary of a different type. While other possible transitions to chaos have been found to be related to a boundary crisis (Arnol'd et al., 1994) as in the Hindmarsh-Rose model (Holden and Fan, 1992), here this question will remain the subject for future work.

The importance of time-scale differences in Hodgkin-Huxley type equations has already been investigated by some authors (Doi et al., 2001; Doi and Kumagai, 2005). In our case, we explored the contribution of different time scales and found that chaos appears only in certain ranges of slow time constants τ_*sd*_, τ_*sr*_ and τ_*h*_. However, in contrast to the mentioned previous works, our model exhibits chaotic behavior within the biologically plausible values of these parameters.

Our findings emerge as an important contribution to the existing literature on the role of homoclinic bifurcations in concrete neuronal models (Feudel et al., 2000; Shilnikov and Cymbalyuk, 2005; Shilnikov, 2012). Future work on the chaotic behavior of the HB+Ih model can include a more detailed geometrical analysis of the chaotic attractors and a deeper investigation of bifurcations occurring at the onset of chaos. Indeed, chaotic dynamics can also be triggered by a wide range of global bifurcations such as homoclinic and heteroclinic phenomena (Aguirre et al., 2013, 2014) which have yet to be analyzed in the HB+Ih model. The simplicity of this model and the fact that its equations and parameters maintain biophysical meaning, can make it a useful tool to understand how chaotic brain dynamics can be shaped by changes in ion channel expression or to give crucial insight to characterize properties of healthy and ill brains.

## Author contributions

KX, JM, MC, and PO performed numerical simulations and analysis. DQ and PO performed bifurcation continuation analysis. KX, JM, PA, and PO wrote the manuscript. KX, JM, MC, DQ, PA, and PO revised and approved the manuscript.

## Funding

This work was funded by Grants Fondecyt 1130862, ACT-1113, and the Advanced Center for Electrical and Electronic Engineering (FB0008 Conicyt, Chile). The Centro Interdisciplinario de Neurociencia de Valparaíso (CINV) is a Millennium Institute supported by the Millennium Scientific Initiative of the Ministerio de Economía (Chile). PA was partially funded by Proyecto Fondecyt Iniciación 11150306.

### Conflict of interest statement

The authors declare that the research was conducted in the absence of any commercial or financial relationships that could be construed as a potential conflict of interest.
